# Women participating in sport: tensions rising from negotiations of aging, gender norms, and personal responsibility for health in later life

**DOI:** 10.3389/fspor.2025.1655912

**Published:** 2025-09-08

**Authors:** Paula M. van Wyk, Hannah Seguin, Rylee A. Dionigi, Patti L. Weir, Sean Horton

**Affiliations:** ^1^ Department of Kinesiology, Faculty of Human Kinetics, University of Windsor, Windsor, ON, Canada; ^2^ WE SPARK, University of Windsor, Windsor, ON, Canada; ^3^ School of Allied Health, Exercise & Sports Sciences, Faculty of Science & Health, Charles Sturt University, Port Macquarie, NSW, Australia

**Keywords:** older age, gender-based constraints, double barrier, qualitative, agency, competitive, recreational

## Abstract

**Introduction:**

Older women have typically faced systemic exclusion from sport, often a result of intersecting age- and gender-based norms and/or constraints. This study investigated how 22 women (mean age 61 years) participating in recreational or competitive sport understood and experienced their participation in relation to societal expectations of aging, gender, and maintaining health and wellbeing.

**Methods:**

The women, aged 52–77 years, each participated in a semi-structured interview to explore their perspectives on aging, disability, societal perceptions, and sport engagement. Interviews were audio-recorded, transcribed, anonymized, and analyzed using reflexive thematic analysis, emphasizing researcher subjectivity and iterative theme development.

**Results:**

The women framed their sport involvement as a moral and disciplined practice, aligning with neoliberal ideals of personal responsibility and self-management for health in later life. However, their narratives also highlighted systemic barriers, such as professional demands, caregiving responsibilities, and gendered norms, that constrained their participation. This “double barrier” of age and gender norms produced a tension between perceived agency and structural exclusion.

**Discussion:**

While older women actively asserted responsibility for their health and engagement, their experiences revealed that structural inequities related to age and gender expectations, not personal failings, often limited participation.

**Conclusion:**

These findings challenge responsibility-centred narratives and call for inclusive sport policies that account for the socio-cultural and institutional barriers shaping older women's experiences in sport and exercise contexts.

## 1 Introduction

Historically, both age and gender act as barriers to sport participation, particularly for older women. Prior to the 1960s, dominant Western discourses framed aging as a period of inevitable physiological decline and characterized appropriate later-life activities as passive, sedentary, and domestic (1). This ageist ideology historically positions the aging body as frail and incapable of exertion, rendering participation in sport socially inappropriate and medically suspect (2), yet today the participation of older people in sport is in the process of being normalized (3). Such contradictory representations of age and gender empower certain groups, typically Caucasian males, and marginalize some older adults from the realm of athleticism, such as people with an age-related disability or women who did not have the opportunity to compete when they were younger, ultimately reinforcing broader cultural narratives of gender norms, and aging as decline and disengagement (4–6), which can influence how age and gender are subjectively understood and experienced by older women today.

This exclusion and stereotyping are compounded for women because they are further marginalized within sporting spaces due to entrenched gender norms privileging male participation and visibility (4, 6, 7). Alongside a limited understanding of the complexities of sport (recreational and competitive) participation among older women (8), this historical marginalization produces a “double barrier”: older women are rendered largely invisible within both sport and scholarly discourse as they contend with intersecting ageist and gendered assumptions (5, 6, 8, 9).

Although during the past half-century there has been a notable increase in sport participation among older populations, including women, this shift is neither ideologically neutral nor universally empowering. Rather, it reflects a broader reconfiguration of aging under neoliberal governance, wherein individuals are increasingly held responsible for maintaining health, productivity, and societal value (3, 10, 11). Contemporary public health and policy discourses now promote lifelong physical activity as a normative and moral obligation, positioning sport as both a panacea for age-related decline (12) and a mechanism for individual “responsibilization” (13, 14). Within this framework, physically active older adults are celebrated as “successful agers” and ideal neoliberal subjects, while those who remain sedentary are pathologized, marginalized, or blamed for their health status (11, 15). This medicalization and moralization of physical activity obscures socio-economic, cultural, and personal constraints shaping individuals' choices, effectively erasing more inclusive and pluralistic understandings of aging and leisure (3, 12).

Accordingly, there is a pressing need for current, nuanced inquiry into the specific constraints and implications of sport participation for older women. Such inquiry must extend beyond descriptive accounts to critically examine how older women navigate intersecting individual and socio-structural barriers, including normative expectations around personal agency and the moral imperative to self-manage health and wellbeing (8). For older women in particular, participation in sport is fraught with contradictions. While sport can offer spaces to resist ageist and sexist ideologies—enabling empowerment, visibility, and agency—it may simultaneously reinforce dominant discourses that marginalize people unable or unwilling to conform (4–6, 16).

This paper critically examines the intersecting discourses of aging, gender, and neoliberalism as they manifest in the narratives and lived experiences of older women engaged in sport. In doing so, it seeks to illuminate the complex ways in which sport functions as both a tool of empowerment and a mechanism of normative regulation in later life in Western culture. This study asks: how do older women involved in recreational or competitive sport understand and experience their participation in relation to societal expectations of aging, gender, and maintaining health and wellbeing?

## 2 Methodology

### 2.1 Positionality statement

As researchers with diverse backgrounds and academic trajectories, the authors bring unique perspectives to this study. Their collective expertise spans aging, sport sociology, leisure studies, physical activity, disability, and social gerontology, enabling a multidimensional understanding of the complexities surrounding older women's experiences in sport. Their positionalities—as scholars who may or may not share the same lived experiences as the participants, and as individuals who have had access to educational and healthcare resources—necessitate a critical awareness of how power dynamics, academic lenses, and disciplinary norms shape the questions asked, the interpretations made, and the narratives privileged. Throughout this work, and particularly during the reflexive thematic analysis process, the authors remain committed to amplifying the voices of older women themselves, striving to conduct research that explores their experiences, while also respecting their embodied knowledge and agency in sport, and acknowledging that our positionality ultimately shapes the researcher-participant relationship.

### 2.2 Participants & data collection

This study draws from interviews with 22 participants who self-identified as female, were aged from 52–77 years (mean = 61 years) and were primarily white and well educated (see Table 1). To be included in the study, the participants had to be at least 50 years of age. Studying women 50 + years is appropriate as this group is commonly defined as “older” in sport-specific research (e.g., 17, 18) and is the minimum age requirement for events like the Huntsman World Senior Games (https://seniorgames.net/about). Participants described their involvement in sport as either recreational (rec) or competitive (comp), examples include tennis, softball, pickleball, powerlifting, running, and dragon boat racing. Given the limited research on older women engaged in recreational sport (8), this study included a broad range of sporting involvement to enable a more comprehensive understanding of how older women experienced and valued sport.

**Table 1:** Participant self-identified demographics.

Pseudonym	Age (y)	Cultural/Ethnic Origin^a^	Education (highest completed)	Occupation^b^	Competitive/Recreational^c^
Florence	68	English	University (bachelor's)	Retired + part-time employed	Competitive
Inez	69	French-Canadian	High school	Retired + volunteer + caregiver	Competitive
Henrietta	68	Anglo-Saxon	University (bachelor's)	Retired + volunteer	Competitive
Alma	54	Italian	University (master's)	Volunteer	Competitive
Ruth	55	White	University (post-graduate)	Part-time employed	Competitive
Yvonne	72	Caucasian	University (bachelor's)	Retired	Competitive
Indira	55	Caucasian-Finnish, Irish, Scottish	University (bachelor's)	Full-time employed	Competitive
Dorothy	77	English, Irish, French-Canadian	High school	Retired + caregiver	Recreational
Constance	67	Caucasian	University (bachelor's)	Retired	Recreational
Mei-Ling	70	Indian	College	Retired	Recreational
Lillian	65	Canadian	College	Retired + volunteer	Recreational
Claudia	63	Caucasian	High school	Retired	Recreational
Francesca	56	Canadian	College	Full-time employed + homemaker	Recreational
Fatima	53	White	University (bachelor's)	Part-time employed	Recreational
Anita	67	Caucasian	University (master's)	Retired	Recreational
Helga	55	British, Northern European	College	Full-time employed	Recreational
Sharon	59	Canadian, Caucasian	University (bachelor's)	Volunteer	Recreational
Catherine	57	Caucasian	College	Full-time employed	Recreational
Zeinab	52	Sri Lankan	University	Full-time employed	Recreational
Agnes	63	Polish, Ukrainian	University (bachelor's)	Full-time employed	Recreational
Nadine	55	White	University (bachelor's)	Full-time employed	Recreational
Betsy	56	Caucasian	University (bachelor's)	Retired + part-time employed	Recreational

To ascertain certain demographic information, participants responded to the following questions.

^a^ What is the ethnic or cultural group that you belong to or identify with?

^b^ Regarding your occupation, which of these describe you (select all that apply)? Choices were Retired, Volunteer, Full-time employed, Part-time employed, Caregiver, Homemaker, Other (open-ended).

^c^ Do you engage in any sport competitively? (“Competitively” as defined by participation in competitions at the local level and beyond or where involvement is geared toward performance and victory).

The research team collaboratively developed the semi-structured interview guide, informed by previous studies related to active aging. The interview questions explored key concepts related to the aging population, disability prevalence in later life, societal perceptions of decline and dependency, rising sport participation among older adults, and the benefits of lifelong sport participation. Following ethics clearance and informed consent, all participants were interviewed once for approximately an hour. All interviews were audio-recorded, transcribed verbatim, and anonymized for further analysis. All participants were assigned a pseudonym.

### 2.3 Data analysis

A reflexive thematic analysis was conducted following the approach outlined by Braun et al. (19), with an emphasis on researcher positionality as a resource in meaning-making. The analytic process began with familiarization, involving repeated reading of transcripts to develop a comprehensive understanding of the data. Initial codes were generated inductively, capturing segments of data relevant to the research aims. Coding was treated as a flexible and interpretive process, enabling the development of rich and nuanced insights.

Codes were refined through iterative engagement with the data, reflecting an evolving understanding of participants' meanings and experiences. Through this process, patterns of shared meaning were identified and organized into preliminary themes that conveyed coherent and conceptually rich stories within the data. Terminology for themes was developed to reflect their central organizing concepts, and ongoing reflexive engagement by the authors, with both the data and analytic decisions, was maintained throughout, in line with the recursive and creative nature of reflexive thematic analysis (19). The resulting themes were interpreted within broader narratives of societal expectations of aging and gender to highlight the contradictory ways in which sport functions as both an instrument for personal empowerment and a structural mechanism of normative regulation for women in later life in Western society.

## 3 Results

The analysis yielded two broad themes: Personal responsibility for health and wellbeing through sport participation, and; Double barrier of age and gender norms to sport participation.

### 3.1 Personal responsibility

A central concept identified across the women's narratives was personal responsibility—specifically, their perceived obligation to maintain their health and wellbeing, through their engagement in sport. The women framed this responsibility as a moral duty, a matter of self-discipline and routine, and an intentional, preventative approach to age-related decline or disability. Remaining physically active was not viewed as optional; it represented a foundational value that shaped their identity, lifestyle, and influences on others.

The women described their engagement in sport and exercise as an essential way to care for their bodies. They viewed sport and exercise participation as beyond being beneficial, that it was a moral obligation. Helga (55, rec) articulated this perspective by emphasizing that neglecting one's body was tantamount to disrespecting a divine gift: “*I can't imagine not being involved in sport. I feel like it's irresponsible. Our bodies and our life are a gift, not maintaining your body is really an insult to God. He doesn't give us anything that we're supposed to be misusing or neglecting. Our bodies are amazing things that we know how to keep them healthy, I would just feel really guilty and irresponsible if I weren't involved in sport”.*

This sense of obligation extended beyond physical health, anchoring participants morally and emotionally. Indira (55, comp) reinforced this point by stating: “*Everybody could move, as long as you're breathing, you should be moving, and when you're moving, you could move to the next stage of movement no matter where you're at”.*

The women emphasized that remaining active required intentionality, consistency, and self-discipline, which is something that organized sport provided for them. Claudia (63, rec) spoke about the role of motivation, observing that retirement provided a space for reflection and renewed commitment: “*I guess you got to motivate, it is the motivation on yourself. When I was working, I wasn't as fit, and as I retired I wanted to become more fit. But you still gotta motivate yourself. That's the key”.*

According to participants, sport and exercise as habits needed to be intentional and cultivated across the lifespan. Fatima (53, rec) suggested that sustained involvement delayed aging and preserved vitality: “*Life without sports? I don't think so. … it's important your whole life to stay involved in sport. If I were to stop here, I would age quicker. My goal is to just continue doing sports as long as I can”.*

In line with this lifelong commitment, participants viewed aging as an active opportunity to invest in their future health. Sometimes this preventive mindset came from paralleling—reflecting on the aging of others, such as parents or peers, as powerful motivators in varying ways. Alma (54, comp) shared how her mother's limited mobility influenced her own preventive mindset: “*As I get older, I see my mom who's 91 and she's very limited in her mobility. I know that as I age, I'm probably going to be able to do less and less but I don't want to get to be 91 and be where my mom is. For me keeping active, doing different things, keeping moving all the time, it's going to help me age better, I hope. That, for me is a huge incentive to keep doing what I'm doing”.*

Perhaps what is most striking about the above quote is that despite her mother being 91 years old, Alma does not accept the prospect of having “*very limited… mobility”* in her 90s, which is part of her motivation for continued sport participation. Similarly, Fatima (53, rec) emphasized: “*You have to make yourself a priority, which I learned as well…I would encourage people to continue to be healthy … some people are still running in their 90s, don't put a timeline on it or don't think that when you reach a certain age, you shouldn't be out there being active with other people”.*

Moreover, many women connected personal responsibility to disease prevention, pain management, and functional preservation, aligning with health promotion narratives in Western cultures. Betsy (56, rec) emphasized the gender-specific relevance of physical activity: “*From a women's health perspective, bone density is super important. I've got a big history of osteoporosis in my family and so I've been doing weight bearing things … like a muscle mass perspective, keeping your core strong to try to avoid spinal problems, postural problems”.*

Likewise, Lillian (65, rec) illustrated how she adapted exercise to her environment to continue with sport: “*I'm always going to have to do something. Even if I run up and down my stairs, a lot of stuff you can do. I mean hold on to the kitchen counter and I lift up my legs. For my balance, do circles with my legs… for my ankles, do rocking for my ankles. … There's a lot of stuff you can do. I would probably do my own little routine”.*

Even when facing physical limitations, these women portrayed a “no excuses” mentality in relation to their age and gender when it came to explaining their ongoing commitment to sport participation. Helga (55, rec) was forthright when stating: “*people need less mollycoddling and excuses”*. This mentality of sport involvement, as a moral and disciplined practice, aligns with neoliberal, Western ideals of personal responsibility and self-management for health in later life. However, their narratives also highlighted systemic barriers, such as age- and gender-related norms and expectations, that constrained their participation.

### 3.2 Double barrier

Although the women emphasized personal responsibility in maintaining health and engagement in sport, they also acknowledged the challenges posed by their intersecting identities as older women. These compounded challenges, referred to in the literature as the “double barrier” [e.g., 8], stemmed from the simultaneous effects of ageism and sexism, which together constrained participants' opportunities, visibility, and societal support for sport participation. Participants articulated these barriers through discussions of life transitions and competing responsibilities (even at younger ages), physical vulnerability, and structural gender inequities. These transitions were not fleeting, often resulting in prolonged periods of withdrawal from sport. This double barrier of age and gender norms produced a tension between perceived agency and structural exclusion in relation to their sport participation.

The women described how professional demands and/or caregiving responsibilities disrupted their engagement in sport. Nadine (55, rec) reflected on how launching a business required her to disengage from previously consistent sport involvement due to concerns about injury and time constraints: “*I remember that from that young age… I've always been involved with track, volleyball, baseball, those types of sports … those all went sort of through high school and young, the early 20s through to mid 20s. But then, I started a business and I really got away from sports … because I was on my own and I was running a business, that I just felt like I couldn't play sports because I didn't want to get hurt”.*

Caregiving responsibilities related to raising children, aging parents, and spouses affected the women's sport participation in mid- and later-life. Childrearing, and caring for aging parents, combined with the emotional labor of managing household responsibilities were key barriers to sport participation, particularly during midlife. Indira (55, comp) explained how the exhaustion of full-time work and parenting made even initiating sport and exercise participation feel daunting: “*When your kids are little, demands are really high, and if you're working full time too, that exhaustion just takes over and it's really hard to pull yourself out. The hardest step is getting out of the door”.*

Mei-Ling (70, rec) added that caregiving remained a lifelong responsibility, as she often put aside her own needs to assist her children in mid-life, and then, as an older adult, her grandchildren. She described this as a deeply embedded sense of duty: “*If my son calls me and says [grandchild] is not feeling well and I am busy at work, would you mind going and getting her? I will drop every single thing. It's very important, that my children and grandchildren, be attended to”.*

The caregiving burden described above extended across generations for many women. Lillian (65, rec) further explained the competing responsibilities: “*Just having a family. Married. No time for you, meaning me. … And my parents were elderly. Looking back, it was busy. Looking after my parents, and having a family of my own, and working”.*

Dorothy (77, rec), who was caring for a spouse with dementia, further added that to the narrative of lacking time for oneself as an older woman. She noted that the increasing demands on her time made sustaining personal engagement in sport and exercise routines particularly challenging: “*I'm a caregiver, right? For my husband who has dementia, the time factor, it's difficult to make time for myself”*. Although 20 years younger than Dorothy, Betsy (56, rec) also highlighted the wants of her husband were prioritized before her own needs: “*The fact that my husband was training at a fairly high level also meant that I was doing more with the kids, right? That all contributed to time being a bit of a barrier”.*

The women also reflected on how physical vulnerability, shaped by both internal bodily changes and external structural limitations associated with age and gender, impacted their sport engagement. Participants spoke of growing awareness of their bodies' limitations and described needing to adjust their physical routines accordingly. Menopause was an emphasized factor that reshaped participants' physical capacity and motivation for sport participation. Alma (54, comp) recognized a marked decline in both physical performance and emotional wellbeing during this transition: *“Menopause … that has definitely impacted what I'm able to do. It's kind of hit me like I ran into a wall when that hit and it is all kinds of things, from physically slowing down a lot—to the anxiety of anything, even practice, that I never had before. Menopause sucked … it definitely had an impact on my performance”.*

As Francesca (56, rec) noted: “*it's hard to say because I don't know if it's getting older or it's menopause,”* highlighting how participants found it difficult to disentangle whether the changes they were experiencing were due to aging or menopause. Nonetheless, all of the women agreed that these physiological shifts presented significant hurdles that they were determined to manage so that they could continue participation in sport and exercise.

In addition to physiological barriers, participants identified structural gender inequities that shaped both their early and current sport experiences. Claudia (63, rec) noted a sense of discomfort and exclusion in male-dominated sport environments, where she encountered aggression and a lack of inclusivity: *“I don't want to play with the men. They are fun and all that, but they can be kind of nasty,”* she explained.

The women shared how throughout their lives gender norms influenced sport selection and perceived safety while participating. Agnes (63, rec) recalled how her parents steered her toward figure skating while her brothers played hockey: “*When I was about 10 years old, getting involved in sport, my brothers were playing hockey. I had three brothers, they were all playing hockey. My parents were always at the arena, I was at the arena, and I just felt kind of left out. So, my mother got me involved in figure skating. I did that when I was in my teens, until I went to university”.*

Societal expectations related to appearance, gender, and aging norms further shaped participants' experiences with sport. The women said that they would receive dismissive or gendered comments by others if the sport they participated in was traditionally associated with males and gendered norms tied to men. For example, Indira (55, comp) shared: “*I do kind of get my back up, especially with some of the younger women at the gym, who will say openly to me, ‘oh, well, I don't want to look like a man or I don't want to lift heavy’ because they don't want to train like a man. They don't want to look like men. The implication is that that's what I do, you know”.*

Reflecting an ongoing Western cultural preoccupation with bodily appearance, even in older age, and especially for females, Nadine (55, rec) shared how others commented on her weight loss through playing pickleball: “*It's amazing how many people said to me, ‘wow, you've lost a lot of weight playing pickleball.’ I don't know if it's all from that. I certainly have had to be watching what I'm eating so, being more conscious of that. The weight part of it is, I guess that can be at any age, although probably age ends up in there. I think going through menopause and that, it's harder to lose weight”.*

Nadine adds another layer of complexity to her sport participation by tying her difficulties with weight management back to the realities of hormonal changes in menopause. Simultaneously, there were some women who expressed pride in their resilience and advocated for greater recognition of middle-aged and older women's contributions to sport. Alma's (54, comp) statement captured this sentiment powerfully: “*We're the biggest section of the Canadian population. Pay attention to us now. And I think that as women, we are strong. We can make ourselves heard and seen, and we can influence change”.*

The above quote captures the tension between perceived personal agency and structural exclusion that this “double barrier” of age and gender norms produced. While these women actively asserted responsibility for their health and engagement, their narratives also highlighted systemic barriers, societal norms, and biological realties (e.g., menopause) related to age and gender, more so than personal failings, that often limited their sport participation.

## 4 Discussion

This study explored how middle-aged and older women engaged with sport and exercise across recreational and competitive contexts, revealing that personal responsibility operated as a central, enduring principle throughout their lives. Participants framed their involvement in sport as a moral practice—an expression of self-discipline and daily commitment to personal health, adaptability, and accountability. They positioned themselves as active agents in maintaining their health and motivation to be physically active, aligning with neoliberal discourses that frame aging and wellness as matters of individual choice, self-management, and moral responsibility, as has also been found among older athletes in competitive sport more generally (3, 11, 13, 14). Within these discourses, the aging body becomes a site of personal investment, and aging well is measured through visible activity and engagement. Adding to this complexity are gender norms, social expectations, and physiological realities related to being an older woman participating in sport, which are the unique contributions that our study brought to the fore.

The women's emphasis on personal responsibility reflected a broader neoliberal, Western ideology that shifts accountability from structural systems onto individuals (10, 15). By privileging discipline, self-regulation, and autonomy, this perspective obscured the social and institutional conditions shaping access to sport and physical activity (12). While neoliberal narratives may encourage participation among some, typically Caucasian, middle-class older adults (3), they risk positioning inactivity as a moral failure, ignoring the impact of intersecting social determinants and identity-based exclusions, such as age, gender, race, and class (11).

Shifting the narrative to name, blame, and shame older women for physical inactivity fails to account for the explanations, not excuses, that are legitimate. The women's accounts revealed tensions between individual agency and structural inequities. While some insisted they had “no excuses” for inactivity, others explained structural constraints such as menopause, caregiving responsibilities, and gendered stereotypes. This tension underscored the complex intersection of aging, gender, and health discourses that expect women to demonstrate personal agency through their ongoing sport participation while negotiating social, biological, and institutional realities and inequities. These findings call for caution in advancing responsibility-based narratives that erase or overlook the socio-cultural and institutional exclusions related to later life sport participation for women. Without attending to these broader issues, such narratives risk reinforcing marginalization by blaming individuals for their non-participation in systems that exclude them (11, 15).

The women's narratives illustrated how personal responsibility operated within the age- and gender-based constraints of a double barrier (8). While societal gender expectations may be different for younger and middle-aged women, compared to older women, these expectations or perceived responsibilities were barriers that these women had to negotiate across their lifecourse. Navigating these tensions required continuous negotiation between the women's personal aspirations and competing demands of professional obligations, caregiving, and societal gender roles. Despite strong agency, the women encountered sport environments that were not designed with their needs in mind. They described gendered assumptions that undermined their legitimacy as athletes, and external obligations (e.g., caregiving, employment) that restricted their time and energy for sport. These findings aligned with literature highlighting how sport participation and physical activity for older women are shaped and constrained by broader social factors and constructions of gender (4, 5, 7, 16, 20).

The juxtaposition of neoliberal discourses and the double barrier of age and gender revealed a central tension: while women were encouraged to take responsibility for their own aging through regular sport and exercise participation, structural conditions frequently impeded such efforts. Neoliberalism functioned to mask these inequities by individualizing responsibility for health and participation, thereby obscuring the age- and gender-based discrimination embedded in many sport contexts (3, 14, 16). The women described reductions in visibility, legitimacy, and access within sport that reinforced their exclusion through both social norms and institutional practices. These age- and gender-based barriers were not incidental but systemic, shaped by long-standing inequities that continue to disadvantage older women in sport and exercise settings, despite these women being primarily from Caucasian and relatively high educational backgrounds. Given these findings, further research is needed on women of diverse backgrounds in relation to sport and physical activity, which would lend itself to examine this phenomenon through an intersectional lens to account for how overlapping identifies (e.g., age, gender, race, etc.) structure sport participation and experiences of exclusion. Additionally, drawing parallels with high-performance athletes may further highlight how common tensions, such as discipline vs. constraint or agency vs. structural limitation, are lived differently across life stages and sporting contexts. Comparative, intergenerational analyses could also extend this work by examining how societal expectations around aging and gender vary across cohorts, thereby clarifying how lived experiences shift across the lifecourse and informing broader theoretical and policy implications.

Overall, these findings emphasized the need to critically interrogate responsibility-focused discourses that dominate narratives of aging and sport. While personal agency is important, it must be understood within the broader context of structural constraint. Promoting equitable sport engagement requires moving beyond neoliberal framings toward inclusive, context-sensitive approaches that address material barriers and challenge cultural narratives that equate sport and exercise with moral virtue and ignore the complex realities of aging in gendered and unequal systems.

## 5 Conclusion

The intersection of neoliberalism and the double barrier of age and gender highlighted a core tension between personal responsibility and structural inequality in older women's sport participation. Neoliberal ideologies framed health and wellbeing as individual responsibilities, promoting personal agency while obscuring systemic age- and gender-based constraints (3, 10, 13). These intersecting forces created a double barrier, marginalizing women based on both age and gender and reducing their visibility and legitimacy in sport contexts.

The findings of this study underscored a fundamental paradox: while older women were encouraged, and often internalize the expectation, to take personal responsibility for aging well through sport, their capacity was constrained by persistent structural inequities. This contradiction exposed the inadequacy of individualistic frameworks in addressing the complex realities of aging and gender. This study called for a shift toward equity-focused, structurally informed approaches that challenge simplistic responsibility narratives and address the socio-cultural and institutional barriers shaping participation. Only through such a shift can sport become a more inclusive and supportive space for older women across all levels of engagement.

## Data Availability

The raw data supporting the conclusions of this article will be made available by the authors, without undue reservation.
